# Polyphenolic Compositions and Chromatic Characteristics of Bog Bilberry Syrup Wines

**DOI:** 10.3390/molecules201119662

**Published:** 2015-11-04

**Authors:** Shu-Xun Liu, Hang-Yu Yang, Si-Yu Li, Jia-Yue Zhang, Teng Li, Bao-Qing Zhu, Bo-Lin Zhang

**Affiliations:** 1 Beijing Key Laboratory of Forestry Food Processing and Safety, Department of Food science, College of Biological Sciences and Biotechnology, Beijing Forestry University, Beijing 10083, China; liushuxun@bjfu.edu.cn (S.-X.L.); yanghangyu2015@163.com (H.-Y.Y.); zjiayue1011@163.com (J.-Y.Z.); liteng24@126.com (T.L.); 2 Center for Viticulture and Enology, College of Food Science & Nutritional Engineering, China Agricultural University, Beijing 100083, China; mnblsy@163.com

**Keywords:** bog bilberry syrup wine, polyphenolic composition, chromatic characteristics, bottle aging

## Abstract

Phenolic compounds determine the color quality of fruit wines. In this study, the phenolic compound content and composition, color characteristics and changes during 6 months of bottle aging were studied in wines fermented with bog bilberry syrup under three different pHs. The total anthocyanins and total phenols were around 15.12–16.23 mg/L and 475.82 to 486.50 mg GAE/L in fresh wines and declined 22%–31% and about 11% in bottle aged wines, respectively. In fresh wines, eight anthocyanins, six phenolic aids and 14 flavonols, but no flavon-3-ols were identified; Malvidin-3-*O*-glucoside, petunidin-3-*O*-glucoside and delphinium-3-*O*-glucoside were the predominant pigments; Chlorogentic acid was the most abundant phenolic acid, and quercetin-3-*O*-galactoside and myricetin-3-*O*-galactoside accounted for nearly 90% of the total flavonols. During 6 months of bottle storage, the amounts of all the monomeric anthocyanins and phenolic acids were reduced dramatically, while the glycosidyl flavonols remained constant or were less reduced and their corresponding aglycones increased a lot. The effects of aging on blueberry wine color were described as the loss of color intensity with a dramatic change in color hue, from initial red-purple up to final red-brick nuances, while the pH of the fermentation matrix was negatively related to the color stability of aged wine.

## 1. Introduction

*Vaccinium uliginosum*, known as bog bilberry, belongs to the *Ericaceae* family of the *Vaccinium* genus [1]. It is one of the most abundant wild blueberries in the Greater Khingan Range, Northeast China. *V. uliginosum* berries are not only rich in monosaccharides, amino acids, dietary fiber, vitamins and trace elements such as potassium, iron, zinc and manganese [1], but also contain a variety and high contents of bioactive substances, including anthocyanins and flavonols [2,3,4,5,6]. During past years, it was well documented that *V. uliginosum* berries have several kinds of high nutritional values and medicinal effects, such as preventing cranial nerve aging, strengthening cardiac functions, combating cancers, softening blood vessels and enhancing immunity [7,8,9,10].

Due to their low sugars and high organic acids contents, *V. uliginosum* berries are not consumed fresh but rather usually steeped in a solution with high sugar content for hours and then dried into fruit snacks for local markets. During the soaking process, some berries are unavoidably crushed due to their quite thin skins. The sugar solutions are then turned into blueberry syrup as by-products after the snack batch production cycles. The related syrup, which not only contain a high content of sugars, but also have considerable amounts of organic acids and polyphenols, are an ideal raw material for fruit wine production [11,12]. Our research groups have developed a process to convert these syrup into fermented red wines by supplementation of an appropriate nitrogen source such as dibasic ammonium phosphate (DAP) [13].

The colors matter a lot for the sensory perception of red wines when the consumers make choices. Anthocyanins, which give young red wines their typical purple red color and decide their color intensity, are the most important phenolic substances, but they are also unstable during the enology process. Previous studies on grape wines found a progressive loss of anthocyanins during the winemaking and aging process, which can be caused by different mechanisms including co-pigmentation, cycloaddition, polymerization or oxidation [14]. It is well known that these reactions are relevant to both the molecular structure of anthocyanins and the matrix of a specific wine. Most of the previous research about the relationships between chromatic characteristics and chemical composition were focused on red grape wines [15,16,17,18]. The anthocyanin, flavonol and organic acid composition of bog bilberries is well documented and distinct from that of wine grapes [2,3,4,5,6]. However, currently, there is a lack of studies regarding the color stability and phenolic compound changes of wines fermented with non-grape fruits, including blueberries. 

This research aims to figure out: (1) the content, composition and changes of anthocyanins and other polyphenols in wines fermented from bog bilberries syrup; (2) the influences of fermentation pH and bottle aging on their color stability.

## 2. Results and Discussion

### 2.1. Conventional Analysis of Blueberry Wines Fermented with Bog Bilberry Syrup under Different pHs

As described previously, the wild blueberry syrup, which are the byproducts of dried berry snack processing and contain plenty of sugars, organic acids and phenolic compounds, were used as raw materials to produce fruit wines by dilution and pH adjustment. Finally, three kinds of wines fermented under three different pH conditions (3.1, 3.3 and 3.5) were obtained and named Wine A, Wine B and Wine C, respectively.

As shown in Table 1, pH had no obvious effect on the alcoholic strength of the final blueberry wines, while Wine B had the lowest total sugar and reducing sugar content, indicating that a pH 3.3 environment was more conducive for yeast to use the sugars in blueberry syrup. The pH of all the resulting wines decreased during the fermentation process, but still showed a certain difference between the different treatments.

**Table 1:** Physicochemical indexes of bog bilberry syrup wines fermented under different pHs.

Oenological Parameters	Wine A	Wine B	Wine C
Fermentation Days *	14	12	13
Alcoholic Strength (% vol)	11.2 ± 0.1 a	11.3 ± 0.3 a	11.2 ± 0.1 a
Total Sugar (g/L)	9.15 ± 0.13 a	7.78 ± 0.39 b	8.74 ± 0.43 a
Reducing Sugar (g/L)	7.1 ± 0.44 a	5.49 ± 0.22 b	5.6 ± 0.02 b
pH (end of fermentation)	3.03 ± 0.01 c	3.21 ± 0.02 b	3.28 ± 0.01 a
Total Acids (g/L)	9.47 ± 0.10 a	9.08 ± 0.20 ab	8.92 ± 0.33 b

Note: * from the start of fermentation to when fermentation naturally terminated; when the relative density dropped to 0.990 and did not change for three consecutive days it was considered the end of fermentation; Different letters in each row indicate the significant differences in the mean at *p* < 0.05.

### 2.2. Changes of Total Anthocyanin and Total Phenol Content in Bog Bilberry Syrup Wines with Different Treatments during the Aging Process

Anthocyanins are mostly responsible for the characteristic red-purplish colors presented by young wines [19], while other polyphenols can stabilize the wine color through co-pigmentation or protecting the anthocyanins by competitively reacting with O_2_ [20,21]. There are a number of studies on the changes of anthocyanins and other polyphenols during the grape wine aging process [16,22,23], but nearly none focused on wines made from other fruits with distinct phenolic compositions. We compared both the total anthocyanin (TA) and total phenol (TP) content in young (after fermentation) and aged (after 6 months bottle storage) blueberries wines.

**Figure 1:**
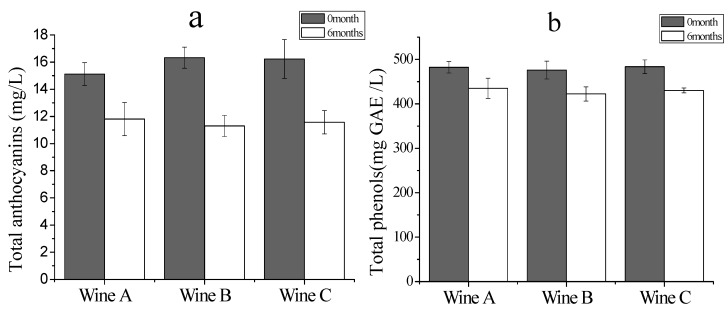
Total anthocyanins (**a**) and total phenols (**b**) of fresh (0 month) and bottle-aged (6 months) bog bilberry syrup wines.

As shown in Figure 1, after fermentation, the contents of TA in Wine A-0M, Wine B-0M and Wine C-0M samples were 15.12, 16.32 and 16.23mg/L, respectively; a little higher than rose grape wines (about 11 mg/L) [24], but much lower than common red grape wines (ranging from 127 to 358 mg/L) [22,23].

As usually reported, the anthocyanins originating from similar berries (such as strawberries, elderberry, and also grape berries) were quite unstable and their degradation rates depended on the pH, temperature and O_2_ concentration of the processing and storage conditions [21,25,26]. TA content decreased considerably during bottle storage, independently of wine type [20,27]. After 6 months of bottle storage, the TA contents in Wine A-6M, B-6M and C-6M declined 21.96%, 30.02% and 30.76%, respectively, which were comparable to rose grape wines (34.5%, from 11.00 mg/L to 7.21 mg/L) [24], but lower than sweet red grape wines (79.75%, from 186 mg/L to 37.7 mg/L) [22] and dry red grape wines (50.15%, from 358.26 mg/L to 178.61 mg/L) [23]. Anthocyanins might undergo complex reactions during aging, among which the oxidative degradation [28] and the formation of insoluble polymeric pigments are the most relevant to the loss of TA and color strength in red wines [15,29].

The total phenols (TP) in our fresh wines ranged from 475.82 to 486.50 mg GAE/L, similar to white grape wines (240 to 500 mg /L), but less than in red grape wines 700 and 4095 mg/L [30]. Over 6 months of aging, the TP in all the samples decreased around 11%, slower than in the white grape wines (about 17%) [31]. In red grape wines, a 15%–22% drop was found during 3 months of bottle aging [16].

It has been proved that the predominant reasons for the decline in TA and TP during wine aging are mainly: (1) oxidative degradation; (2) the polymerization of polyphenolic compounds themselves or reactions with other small compounds such as acetaldehyde and pyruvic acid [32,33,34]. The polymerization reactions result in a more stable color and a better taste and organoleptic quality [33,34], while the irreversible decrease in some bigger polymer pigments, together with the oxidation of monomeric pigments, would make the color intensity decrease [26,35]. The phenolic composition differences between bog bilberry syrup wines and grape wines, which are associated with these chemical reactions occurring along with aging, is valuable to expound.

### 2.3. Comparison of Phenolic Compositions in the Young and Aged Bog Bilberries Syrup Wines

HPLC-ESI-Trap-MS^n^ coupled with a DAD detector was applied to elaborate the anthocyanin and non-anthocyanin phenolic composition of bog bilberry syrup wines and the results are shown in Table 2.

**Table 2:** The concentrations of polyphenolic compounds (mg/L) in bog bilberry syrup wines.

Compound	Months					
	0 Month			6 Months		
	Wine A	Wine B	Wine C	Wine A	Wine B	Wine C
**Anthocyanin**						
Delphinidin-3-*O*-glucoside	2.76 ± 0.13 a	2.51 ± 0.14 abc	2.64 ± 0.21 ab	1.95 ± 0.15 bcd	1.81 ± 0.13 cd	1.76 ± 0.27 d
Cyandin-3-*O*-glucoside	0.36 ± 0.02 a	0.27 ± 0.04 ab	0.27 ± 0.08 ab	0.28 ± 0.00 ab	0.19 ± 0.01 b	0.24 ± 0.04 ab
Petunidin-3-*O*-glucoside	2.97 ± 0.21 a	2.82 ± 0.06 a	3.05 ± 0.26 a	2.21 ± 0.05 b	2.04 ± 0.11 b	2.09 ± 0.01 b
Peonidin-3-*O*-glucoside	0.75 ± 0.04 a	0.68 ± 0.02 a	0.67 ± 0.01 a	0.54 ± 0.02 b	0.48 ± 0.02 b	0.51 ± 0.02 b
Malvidin-3-*O*-glucoside	6.17 ± 0.25 a	6.07 ± 0.05 a	6.14 ± 0.31 a	4.38 ± 0.23 b	4.12 ± 0.24 b	4.34 ± 0.12 b
Malvidin-3-*O*-arabinoside	0.23 ± 0.01 a	0.22 ± 0.01 a	0.21 ± 0.02 ab	0.17 ± 0.00 bc	0.13 ± 0.00 c	0.13 ± 0.02 c
Malvidin-3-(6-acetyl)-glucoside	Tr	Tr	Tr	Tr	Tr	Tr
Malvidin-3-(6-*p*-coumaroyl)-glucoside	Tr	Tr	Tr	Tr	Tr	Tr
**Phenolic acids**						
Gallic acid	Tr	Tr	Tr	Tr	Tr	Tr
Protocatechuic acid	2.89 ± 0.04 ab	2.91 ± 0.1 ab	3.56 ± 0.5 a	1.81 ± 0.08 b	1.77 ± 0.01 b	1.77 ± 0.02 b
Chlorogenic acid	7.15 ± 1.1 ab	7.43 ± 0.11 ab	9.02 ± 1.24 a	5.93 ± 0 b	5.45 ± 0.07 b	5.26 ± 0.57 b
Caffeic acid	0.13 ± 0.05 b	0.29 ± 0.03 ab	0.66 ± 0.27 a	Tr	0.06 ± 0 b	0.06 ± 0.06 b
4-Hydroxycinnamic acid	Tr	Tr	Tr	Tr	Tr	Tr
Salicylic acid	0.2 ± 0 a	0.18 ± 0.01 a	0.24 ± 0.04 a	Tr	Tr	Tr
**Flavonols**						
Myricetin-3-*O*-galactoside	32.64 ± 0.52 a	28.12 ± 0.66 b	28.78 ± 1.28 ab	30.45 ± 1.45 ab	29.41 ± 0.41 ab	29.8 ± 1.07 ab
Myricetin-3-*O*-glucoside	0.8 ± 0.09 a	0.72 ± 0.1 a	0.61 ± 0.08 ab	0.53 ± 0.08 ab	0.37 ± 0.07 b	0.36 ± 0.01 b
Quercetin-3-*O*-galactoside	44.2 ± 1.81 a	39.21 ± 0.8 ab	38.54 ± 1.74 b	38.05 ± 1.69 b	36.93 ± 0.66 b	37.28 ± 1.12 b
Quercetin-3-*O*-glucuronide	2.13 ± 0.16 bc	2.32 ± 0.16 b	1.58 ± 0.07 c	3.13 ± 0.2 ab	3.11 ± 0.08 a	3.15 ± 0.17 a
Quercetin-3-*O*-glucoside	0.67 ± 0.08 a	0.5 ± 0.2 a	0.85 ± 0.44 a	0.49 ± 0.09 a	0.4 ± 0.09 a	0.44 ± 0.05 a
Quercetin-3-*O*-rhamnoside	Tr	Tr	Tr	Tr	Tr	Tr
Syringetin-3-*O*-galactoside	3.94 ± 0.32 a	3.64 ± 0.32 a	4.5 ± 0.7 a	3.47 ± 0.09 a	3.35 ± 0.11 a	3.44 ± 0.06 a
Syringetin-3-*O*-glucoside	0.89 ± 0.1 a	0.91 ± 0.06 a	1.12 ± 0.3 a	0.98 ± 0.15 a	0.82 ± 0.04 a	0.79 ± 0.1 a
Kaempferol-3-*O*-glucoside	Tr	Tr	Tr	Tr	Tr	Tr
Kaempferol-3-*O*-galactoside	Tr	Tr	Tr	Tr	Tr	Tr
Isorhamnetin-3-*O*-glucoside	Tr	Tr	Tr	Tr	Tr	Tr
Myricetin	Nd	Nd	Nd	1.61 ± 0.14 a	1.55 ± 0.02 a	1.51 ± 0.1 a
Quercetin	Nd	Tr	Tr	9.51 ± 1.22 a	9.5 ± 0.34 a	9.4 ± 0.15 a
Syringetin	Tr	Tr	Tr	0.19 ± 0.01 a	0.17 ± 0.01 a	0.19 ± 0.01 a
**Flavan-3-ols**						
Gallocatechin	Nd	Nd	Nd	Nd	Nd	Nd
(−)-Epicatechin	Nd	Nd	Nd	Nd	Nd	Nd
(+)-Catechin	Nd	Nd	Nd	Nd	Nd	Nd
Epigallocatechin gallate	Nd	Nd	Nd	Nd	Nd	Nd

Note: Nd, “Not-Detected”; Tr, “Trace”; Different letters in each row indicate the significant differences in the mean at *p* < 0.05.

#### 2.3.1. Anthocyanins

Six kinds of monomer anthocyanins including delphinidin-3-*O*-glucoside, cyandin-3-*O*-glucoside, petunidin-3-*O*-glucoside, peonidin-3-*O*-glucoside, malvidin-3-*O*-glucoside and malvidin-3-*O*-arabinoside were identified. Two more acylated anthocyanins (3-*O*-acetylglucoside-Mv and 3-*O*-coumaroyl-glucoside-Mv) were detected in all the wine samples, but in trace amounts. The type of anthocyanins, especially the pyranoanthocyanins and acylated anthocyanins, was far less than in red grape wines [36], and even less than in rose grape wines, which had comparable TA contents to the samples studied here [24].

In all the samples, malvidin-3-*O*-glucoside was the predominant pigment, accounting for about 40% of the total anthocyanin content, followed by petunidin-3-*O*-glucoside (about 18%) and delphinium-3-*O*-glucoside (about 16%). This was similar to the previously found monomeric anthocyanin composition of bog bilberries [2,3]. The proportions of malvidin-3-*O*-glucoside (with maximum numbers of methoxy groups on the B rings) were less than in grape wines (about 60%) [16,20], while the proportions of delphinidin-3-*O*-glucoside and petunidin-3-*O*-glucoside, which have more hydroxyl groups and are less stable compared to malvidin-3-*O*-glucoside, were more in our wines than in grape wines (which ranged from 2.11% to 3.41% and 2.64% to 6.34%, respectively) [17,20,36,37]. The numbers of hydroxyl groups on the B ring of a specific anthocyanin is related with its color hue and stability [38]. These differences in anthocyanin composition would make the blueberry wines display a more blue color hue, but less color stability.

During 6 months of bottle storage, all six of the monomeric anthocyanins detected decreased significantly, and it was speculated that they degraded into small phenolic acids and anthocyanone A through oxidation and this finally reduced the related wine color intensity [28], or they changed into more stable oligomers and polymers of pigments through direct and acetaldehyde-mediated anthocyanin-flavanol condensation reactions or other anthocyanin condensation reactions leading to pyranoanthocyanins [18,36,39,40], which could lead to the wine color hue changing from bright-red to brick-red [33]. Although the polymerization products of anthocyanins are quite commonly identified in red grape wines [35,36], they were not detected in our aged blueberiy wines, which might related to their low contents of monomeric anthocyanins. 

#### 2.3.2. Phenolic Acids

A total of six phenolic acids were found in all wine samples (Table 2). Among them, chlorogentic acid was the most abundant phenolic acid, and followed by protocatechuic acid, caffeic acid and salicylic acid (also called 2-hydroxybenzoic acid). Both 4-hydroxycinniamic acid and gallic acid were identified in all our wine samples, but under their limit of quantitation (LOQ). It was also worth noting that some other phenolic acids and their esters, including fertaric acid, coutaric acid and caftaric acid which are quite common in grape wines, were not detected in the wine samples studied here. These results demonstrated that the phenolic acid compositions of blueberry wines were quite distinct from those of grape wines which have been studied in detail previously [16,19,24,41,42]. 

Chlorogentic acid, the ester of caffeic acid and quinic acid, was previously found with a content of 20 mg/L in bog bilberry juice [6]. Its concentration was around 7.15–9.02 mg/L and with no significant differences in our original wines. Chlorogentic acid was also identified in wines made from other fruits including cherries (1.1–21.3 mg/L) [43,44,45,46], peaches (3.59 mg/L) [47] and apples (8.27 mg/L) [48] and found to be a major contributor to the characteristic taste of cherry wine [43]. To our best knowledge, this compound has not been reported in grape wines yet, although a similar compound, caftaric acid (an ester of caffeic acid and tartaric acid), is quite common in fresh grape wines. Plenty of studies have shown that caftaric acid was unstable during bottle aging, independently of the wine type [20,27]. Similarly, we observed a 17%–42% decrease of chlorogentic acid during the bottle aging periods and the proportion of the decline was related to the pH values of the original wines. This might be the first report on the fate of chlorogentic acid in fruit wines during aging. 

The concentrations of protocatechuic acid and salicylic acid in all our fresh wines, were around 3 mg/L and 0.2 mg/L respectively, without any significant differences. Protocatechuic acid was found at the range of 1.3–2.4 mg/L in sweet grape wines [41,49] and 6–7 mg/L in young red wines [16]. The caffeic acid contents in our fresh wines were as follows: Wine A-0M (0.13 mg/L) < Wine B-0M (0.29 mg/L) < Wine C-0M (0.66 mg/L), far less than in red grape wines (4.7–18 mg/L) [16,30], and even less than in rose grape wine (around 1 mg/L) [24].

After 6 months of bottle aging, the levels of all these quantitative phenolic acids were reduced dramatically, which might result from oxidation to the corresponding quinones or polymerization with monomeric anthocyanins to form complex pigment polymers [50,51]. In grape wines, protocatechuic acid and salicylic acid levels were also found to be reduced with bottle aging; while contrarily, caffeic acid levels increase over this period because of the hydrolysis of caftaric acid [23,24,27,52]. 

Many studies have proved that phenolic acids could serve as cofactors of co-pigmentation to stabilize the flavylium cation chromophore of monomeric anthocyanins and thus enhance the red color intensity and bluish hues of young red wines [16,20,38,53].

#### 2.3.3. Flavonols

Previously studies showed that flavonol compounds could also be involved in the co-pigmentation reactions with anthocyanins and might play an important role in stabilizing wine color [20,23,54]. As shown in Table 2, a total of 14 kinds of flavonol compounds were identified in the samples studied here, including 11 flavonol glycosides (five glucosides, four galactosides, one glucuronide and one rhamnoside) and three free flavonols (quercetin, syringetin and myricetin). 

In the fresh wines, only two free flavonols were detected, but their contents were at trace levels. Quercetin-3-*O*-galactoside and myricetin-3-*O*-galactoside were the most abundant glycosides-bound flavonols (accounting for around 51% and 38% of the total flavonols, respectively), followed by syringetin-3-*O*-galactoside, quercetin-3-*O*-glucuronide, syringetin-3-glucoside, quercetin-3-*O*-glucoside and myricetin-3-*O*-glucoside. It was noteworthy that the galactoside-type compound contents were much higher than those of the glucoside-type, in line with the flavonol composition of bog bilberries found previously [3,55], but contrary to the grape and grape wines in which the glucosides were dominant [20,54].

After 6 months of storage, the contents of free flavonols (myricetin, quercetinand syringetin) increased significantly; meanwhile, inversely, the concentrations of most of the flavonol glycosides (except for quercetin-3-*O*-glucuronide) decreased. This resulted from the hydrolysis of the glycosylated derivatives to aglycones during the wine storage time, as previously observed during bottle aging of grape wines [20,22,23]. The changing proportions of flavonol glycosides were quite lower than those of other types of phenolic compounds (anthocyanadins and phenolic acids), which indicate the former are the most stable phenolic components during wine aging.

Interestingly, bog bilberries were found to have the capacity to accumulate amounts of flavon-3-ols [56] during berry development; but unexpectedly, none of the monomeric flavon-3-ols (catechin, epicatechin, gallocatechin and epigallocatechin gallate) were detected in both the young and aged blueberry syrup wines. The method used in this study was originally developed for grape wines and has been applied to the analysis of hundreds of samples and showed good sensitives for these compounds [36], so we can be sure that the lack of flavon-3-ol compounds (not due to any limitation of the instrumental method but to the samples themselves) was another critical distinction between wines fermented with the bog bilberry syrup and wine grapes. It has been well documented that flavon-3-ol compounds not only contribute to the astringent mouth feel and serve as antioxidants during storage, but also could form unstable non-covalent co-pigments with monomeric anthocyanidins in young red grape wines, or finally changed into stable covalently connected complexes in aged wines, both of which are beneficial to the color stability of aging wine [35,38,39,40]. Without flavon-3-ol compounds, the monomeric anthocyanidins was deduced to more easily degrade into small and colorless compounds through oxidation [28], which further might make the blueberry syrup wines more sensitive to free O_2_ during storage.

### 2.4. Chromatic Characteristics

Wine chromatic characteristics, lightness (L*), red/green component (a*), yellow/blue component (b*), chroma (C*), hue angle (h*) and chromatic differences (ΔE*), were evaluated using the CIELab method and the results are shown in Table 3.

**Table 3:** The color parameters of fresh (0 month) and bottle-aged (6 months) bog bilberry syrup wines.

Months	0 Month			6 Months		
	Wine A	Wine B	Wine C	Wine A	Wine B	Wine C
L*	64.23 ± 0.10 b	56.85 ± 0.30 c	58.30 ± 2.35 c	66.22 ± 1.74 b	64.43 ± 0.28 b	71.02 ± 2.67 a
a*	30.92 ± 0.29 a	29.17 ± 0.22 c	30.10 ± 0.31 b	27.46 ± 0.21 d	25.16 ± 0.19 e	25.26 ± 0.18 e
b*	10.22 ± 0.20 d	13.75 ± 0.19 c	13.79 ± 0.52 c	15.39 ± 0.20 b	18.04 ± 0.83 a	15.97 ± 0.27 b
h*	18.33 ± 0 e	25.21 ± 0 d	24.64 ± 0.01 d	29.22 ± 0 c	35.52 ± 0.02 a	32.09 ± 0.01 b
C*	32.56 ± 0.29 ab	32.25 ± 0.24 b	33.11 ± 0.34 a	31.48 ± 0.24 c	30.97 ± 0.46 c	29.88 ± 0.25 d
ΔE*	——	——	——	6.53	9.59	13.78

Note: Different letters in each row indicate the significant differences in the mean at *p* < 0.05.

The CIELab parameter L* (which ranges from 0 for black to 100 for white) is inversely related to the intensity of color. The L* values of the three fresh blueberry syrup wines were compared to the red grape wines, but lower than rose grape wines [24]. Wine A-0M had a higher L* value than B-0M and C-0M. After 6 months of aging, the L* value of wine A did not change significantly, but in contrast, Wine B and Wine C increased from 56.85 to 64.43 and from 58.30 to 71.02, respectively, which means they lost color intensity.

The a* values of our fresh were around 30, lower than young red grape wines (about 45~55) found previously [57,58]; this may be associated with their different anthocyanin compositions, as described in Section 2.3.1. It was also worth noting that the a* value of Wine A was higher than those of Wine B and Wine C. Anthocyanins exist in two structures that can be mutually interconverted when the pH of wine is below 3.2: the flavylium cation (red) and the quinoidal base (blue); With the rise of pH value, the flavylium cations lose protons forming the quinoidal base, at the same time, the flavylium cation is hydrolyzed into the hemiketal or carbinol pseudo-base (colorless), and the hemiketal or carbinol pseudo-base slowly ring opens into a chalcone (colorless) [38]. After 6 months of storage, the a* values of all the studied wines decreased [A (11.19%), B (13.74%), C (16.07%)], which might mainly be due to the precipitation of insoluble polymeric anthocyanin-derived pigments and/or the oxidative degradation of free anthocyanins [21,22,26,35].

With regard to the yellow-blue color component (b*: positive values, yellow component; negative values, blue component) and h*(hue angle): Wine A-0M had lower b* and h* values than the other two wines, which might result from that the lowest pH of A-0M among the three fresh wines, as described in Section 2.1. The values of b* and h* increased during the 6 months of bottle aging. The evolution of b* and h* values with aging time were associated with the formation of anthocyanin-derived yellow-orange pigments like pyranoanthocyanins, and also due to the oxidation of red wine pigments [18,59].

The chromatic differences (ΔE*) is the distance between two points in a three-dimensional space. Our eyes can discriminate samples if their ΔE* more than 3.0 [60,61]. The ΔE* values of Wine A, B and C between 0 month and 6 months of bottle aging were 6.53, 9.59 and 13.78, respectively, which were all higher than 3.0 and easily recognized by human eyes. It was noteworthy that the ΔE* of Wine C was about twice that of Wine A, showing that the pH of the fermentation matrix was indeed negatively related to the color stability of aged wine.

## 3. Experimental Section

### 3.1. The Fermentation of Bog Bilberry Syrup Wines

The bog bilberry syrups (78.1° Brix, pH 2.88), byproducts of dried berry snack production and provided by Xinganlieshen Original Products Ltd., (Hulunbeier City, Inner Mongolia, China), were diluted with volumes of potable water and used as raw materials for wine fermentation. Next, 300 mg/L of dibasic ammonium phosphate (DAP) were added as nitrogen source and various amounts of sodium hydrogen carbonate were added to adjust the pH to 3.1, 3.3 and 3.5, respectively. After that, a commercial dry yeast (*red fruit*^®^, Enartis Ltd., Novara, Italy) was activated and inoculated and all fermentations were conducted under controlled temperature (20–22 °C). When the relative density did not drop in three consecutive days, 100 mg/L of K_2_S_2_O_7_ were added and the temperature were reduced to 4 °C to stop the fermentation and three kinds of wines were obtained and stored in 100 L sealed stainless steel tanks for further research. For the aging tests, some of the fresh wines were filtered with a membrane (0.45 μm average pore size) and packaged into 750 mL glass bottles (dark green color, Bordeaux type, Changyu Ltd., Yantai, China), sealed with natural corks (45 mm in length) and stored in a dark cellar with a constant temperature of 15 ± 1 °C and a humidity of 80% ± 5% for a period of 6 months. Three bottles of wine for each treatment were used for further analysis.

### 3.2. Standard Chemical Analysis of Wines

The amount of total sugar, reducing sugars, total acidity, pH and alcoholic content (% *v*/*v*) were analyzed according to the methods proposed by the National Standard of the People’s Republic of China (GB/T15038-2006, 2006) [62].

### 3.3. Total Anthocyanin Content Measurement

Total anthocyanin content was determined by using a pH differential method according to the AOAC Official Method as described previously [63], but with some modifications. Two dilutions of the extracts were prepared, one for pH 1.0 using KCl buffer and the other for pH 4.5 using sodium acetate buffer, samples were diluted to a factor of 1:3 using the different buffers. Sample spectral absorbance measurements were read at 521 and 700 nm on a spectrophotometer. The total anthocyanins were expressed as malvidin-3-*O*-glucoside equivalents in milligram per liter using the following equation:

(1) $$\text{Total}\;\text{anthocyanin}\;\text{content}\;\left(\frac{\text{mg}}{L}\right)=\;\frac{A\times \text{MW}\times \text{Df}\times 1000}{\varepsilon \times L}$$

where A = (A_521_ − A_700_)_pH1.0_ − (A_521_ − A_700_)_pH4.5_; MW is 493.2, the molecular weight of malvidin-3-*O*-glucoside (g/mol); Df is the dilution factor; ε denotes the extinction coefficient of malvidin-3-*O*-glucoside [28,000 L × mol^−1^ × cm^−1^]; L is the constant at path-length 1 cm; 1000 is the factor to convert g to mg.

### 3.4. Total Phenol Content Measurement

The total phenol contents were determined using the Folin-Ciocalteu method [42], adapted to a microscale experiment. A total of 790 μL of distilled water, 10 μL of sample dissolved in methanol, and 50 μL of Folin-Ciocalteu reagent were added in an Eppendorf tube and vortexed. After 1 min, 150 μL of sodium carbonate solution (20%) was added and vortexed again and stand at room temperature in the obscurity for 2 h. The absorbance was read at 750 nm, and the total phenol concentration was calculated from calibration curve, using gallic acid as the standard. The results were expressed as mg·L^−1^ gallic acid (GAE).

### 3.5. Determination of Phenolic Compounds

An Agilent-1200 HPLC system equipped with a UV detector and an LC-MSD Trap VL ion-trap mass spectrometer (Agilent Technologies, Santa Clara, CA, USA) via an ESI source, were used to analysis the phenolic composition of the wine samples according to the method published by Gao *et al.*, previously [36] with some modifications. 

For anthocyanins—50 μL of filtered samples (cellulose acetate and nitrocellulose, CAN, 0.45 μm) was injected to the system for quantitative and qualitative analyses. A reversed-phase column (Kromasil C18, 250 × 4.6 mm, 5 μm) was used and the mobile phases were as follows: Solvent A (water:formic:acetonitrile = 92:2:6 [*v*/*v*/*v*]) and solvent B (water: formic: acetonitrile = 44:2:54 [*v*/*v*/*v*]). The flow rate was set at 1 mL/min and the gradient was from 0%–10% B for 1 min, from 10%–25% B for 17 min, 25% B for 2 min, 25%–40% B for 10 min, from 40%–70% B for 5 min, 70%–100% B for 5 min. MS conditions were the same with [36]. The anthocyanins were identified by their order of elution time with respect to malvidin-3-*O*-glucoside and the weights of the molecular ion and the fragment ions compared with standards and references [2,36,37]. For quantitative analyses, the detection wavelength used by the diode array detector (DAD) was 525 nm. The concentration of all anthocyanins was expressed as malvidin-3-*O*-glucoside.

For non-anthocyanin phenolic compounds—100 mL of each wine sample was diluted with an equal volume of pure water and extracted three times with ethyl acetate. The organic phase was collected and evaporated to dryness by a rotary evaporator at 30 °C and re-dissolved in 5 mL methanol (chromatographic grade). 2 μL of filtered methanol solution were injected to the HPLC-MS system and a reversed Zorbax SB-C18 column (3 × 50 mm, 1.8 μm) was used for separation. A gradient consisting of solvent A (pure water with 1% acetic acid) and solvent B (acetonitrile1% acetic acid), was applied at the flow rate of 1.0 mL/min as follows: 0%–5% B for 5 min, 5%–8% B for 5 min, 8%–12% B for 5 min, 12%–18% B for 5 min, 18%–22% B for 2 min, 22%–35% B for 2 min, and 35%–100% B for 4 min. The column temperature was 25 °C. MS conditions: negative ion mode; nebulizer pressure, 35 psi.; dry gas flow, 10 mL/min; dry gas temperature, 325 °C; scans at 100–1500 *m*/*z*. For quantification, the detection wavelength was set at 280 nm and an external standard method were used: flavanols using catechin, flavonol using quercetin, hydroxybenzoic acids using gallic acid; hydroxycinnamic acids using caffeic acid and chlorogentic acid using itself, respectively. All of the standards were dissolved with ethanol (HPLC quality) as stock solution, and then this mixed standard solution was diluted into seven levels in succession with the synthetic model wine solution. Mixed standards of each level were analyzed under the same condition as the samples and calibration curves were obtained with their regression coefficients all above 95%.

### 3.6. Characterisation of Colour Parameters

Wine colour was evaluated by the CIELab space method [64,65,66]. The wine samples were previously passed through 0.45 μm pore size filter (cellulose acetate and nitrocellulose, CAN). Absorbances at 440 nm, 530 nm, and 600 nm were measured through a 20 mm light length path on a UV/Vis Spectrophotometer (UNICO, Shanghai, China). The blank was used by distilled water. The lightness (L*), red/green component (a*), yellow/blue component (b*), chroma (C*), hue angle (h*) and the color differences between wines (ΔE*) using the following equation:

(2) $$\begin{array}{c} L^{*}=116{\left(Y/Y_{0}\right)}^{1/3}-16;\\ a^{*}=500\left[{\left(X/X_{0}\right)}^{1/3}-{\left(Y/Y_{0}\right)}^{1/3}\right];\\ b^{*}=200\left[{\left(Y/Y_{0}\right)}^{1/3}-{\left(Z/Z_{0}\right)}^{1/3}\right];\\ C^{*}={\left(a^{*2}+b^{*2}\right)}^{1/2};\\ h^{*}=\text{arctan}\left(b^{*}/a^{*}\right);\\ \Delta E^{*}={\left(\Delta L^{*2}+\Delta a^{*2}+\Delta b^{*2}\right)}^{1/2} \end{array}$$

where X = 14.172T_440_ + 28.583T_530_ + 52.727T_600_ − 0.462; Y = 9.005T_440_ + 62.965T_530_ + 28.168T_600_ − 0.063; Z = 94.708T_440_ + 15.889T_530_ − 5.233T_600_ + 1.777; X_0_ = 97.29; Y_0_ = 100; Z_0_ = 116.14; T_440_ = 10A_440_; T_530_ = 10A_530_; T_600_ = 10A_600_; A_440_, A_530_, A_600_ were the absorbance at wavelength of 440 nm, 530 nm and 600 nm, respectively.

### 3.7. Data Analysis

Calculation of average and standard deviation were performed using Microsoft Excel 2007 software. IBM SPSS Statistics 20 (IBM, New York, NY, USA) for Windows was used for statistical calculations. The differences were considered to be statistically significant when P < 0.05.

## 4. Conclusion

In summary, we obtained three kinds of blueberry wines fermented with wild bog bilberry syrup under different pHs. Polyphenolic content, composition and the chromatic characteristics of these wines, both at the end of fermentation and after 6 months of bottle aging, were studied. The TAs contents in our blueberry wines were similar to those of rose grape wines, and the TPs were comparable to white wines and rose wines. Distinct compositions of both anthocynidins and non-anthocynidins phenolic compounds in our young wines were found compared to grape wines, especially characterized by the lack of flavan-3-ol compounds. During bottle aging, the polyphenolic contents decreased mainly through oxidative degradation, which induced color fading. The effects of aging on blueberry wine color were described as the loss of color intensity with a change in color hue, from initial red-purple to final red-brick nuances. pH was negatively related to the color stability of related wines. These results might help us understand the color stability mechanism in non-grape red wines and improve the organoleptic quality of related wine products further.

## References

[1] Kim Y.H., Bang C.Y., Won E.K., Kim J.P., Choung S.Y. (2009). Antioxidant activities of *Vaccinium uliginosum* L. Extract and its active components. J. Med. Chem..

[2] Li R., Wang P., Guo Q., Wang Z. (2011). Anthocyanin composition and content of the *Vaccinium uliginosum* berry. Food Chem..

[3] Wang L.J., Su S., Wu J., Du H., Li S.S., Huo J.W., Zhang Y., Wang L.S. (2014). Variation of anthocyanins and flavonols in *Vaccinium uliginosum* berry in lesser Khingan mountains and its antioxidant activity. Food Chem..

[4] Andersen Ø.M. (1987). Anthocyanins in fruits of *Vaccinium uliginosum* L. (bog whortleberry). J. Food Sci..

[5] Kusznierewicz B., Piekarska A., Mrugalska B., Konieczka P., Namieśnik J., Bartoszek A. (2012). Phenolic composition and antioxidant properties of polish blue-berried honeysuckle genotypes by HPLC-DAD-MS, HPLC postcolumn derivatization with ABTS or FC, and TLC with DPPH visualization. J. Agric. Food Chem..

[6] Kraujalytė V., Venskutonis P.R., Pukalskas A., Česonienė L., Daubaras R. (2015). Antioxidant properties, phenolic composition and potentiometric sensor array evaluation of commercial and new blueberry (*Vaccinium corymbosum*) and bog blueberry (*Vaccinium uliginosum*) genotypes. Food Chem..

[7] McAnulty L.S., Collier S.R., Landram M.J., Whittaker D.S., Isaacs S.E., Klemka J.M., Cheek S.L., Arms J.C., McAnulty S.R. (2014). Six weeks daily ingestion of whole blueberry powder increases natural killer cell counts and reduces arterial stiffness in sedentary males and females. Nutr. Res..

[8] Basu A., Du M., Leyva M.J., Sanchez K., Betts N.M., Wu M., Aston C.E., Lyons T.J. (2010). Blueberries decrease cardiovascular risk factors in obese men and women with metabolic syndrome. J. Nutr..

[9] Taverniti V., Fracassetti D., Del Bo C., Lanti C., Minuzzo M., Klimis-Zacas D., Riso P., Guglielmetti S. (2014). Immunomodulatory effect of a wild blueberry anthocyanin-rich extract in human caco-2 intestinal cells. J. Agric. Food Chem..

[10] Rodriguez-Mateos A., Del Pino-Garcia R., George T.W., Vidal-Diez A., Heiss C., Spencer J.P. (2014). Impact of processing on the bioavailability and vascular effects of blueberry (poly)phenols. Mol. Nutr. Food Res..

[11] Coelho E., Vilanova M., Genisheva Z., Oliveira J.M., Teixeira J.A., Domingues L. (2015). Systematic approach for the development of fruit wines from industrially processed fruit concentrates, including optimization of fermentation parameters, chemical characterization and sensory evaluation. LWT-Food Sci. Technol..

[12] Jagtap U.B., Bapat V.A. (2015). Wines from fruits other than grapes: Current status and future prospectus. Food Biol. Sci..

[13] Wei M., Gu P., Li C., Yang H., Liu S., Zhang J., Yan Z., Zhang B., Zhu B. (2014). Determination of 7 organic acids in *Vaccinium uliginosum* products by HPLC. China Brew..

[14] Alcalde-Eon C., Escribano-Bailón M.T., Santos-Buelga C., Rivas-Gonzalo J.C. (2006). Changes in the detailed pigment composition of red wine during maturity and ageing: A comprehensive study. Anal. Chim. Acta.

[15] Brouillard R., Dangles O. (1994). Anthocyanin molecular interactions: The first step in the formation of new pigments during wine aging?. Food Chem..

[16] García-Falcón M.S., Pérez-Lamela C., Martínez-Carballo E., Simal-Gándara J. (2007). Determination of phenolic compounds in wines: Influence of bottle storage of young red wines on their evolution. Food Chem..

[17] Trost K., Golc-Wondra A., Prosek M., Milivojevic L. (2008). Anthocyanin degradation of blueberry-aronia nectar in glass compared with carton during storage. J. Food Sci..

[18] Rentzsch M., Schwarz M., Winterhalter P., Blanco-Vega D., Hermosín-Gutiérrez I. (2010). Survey on the content of vitisin A and hydroxyphenyl-pyranoanthocyanins in tempranillo wines. Food Chem..

[19] Lago-Vanzela E.S., Rebello L.P.G., Ramos A.M., Stringheta P.C., Da-Silva R., García-Romero E., Gómez-Alonso S., Hermosín-Gutiérrez I. (2013). Chromatic characteristics and color-related phenolic composition of Brazilian young red wines made from the hybrid grape cultivar BRS Violeta (“BRS Rúbea”×“IAC 1398–21”). Food Res. Int..

[20] Gutiérrez I.H., Lorenzo E.S.-P., Espinosa A.V. (2005). Phenolic composition and magnitude of copigmentation in young and shortly aged red wines made from the cultivars, Cabernet Sauvignon, Cencibel, and Syrah. Food Chem..

[21] Casati C.B., Baeza R., Sanchez V., Catalano A., López P., Zamora M.C. (2015). Thermal degradation kinetics of monomeric anthocyanins, colour changes and storage effect in elderberry juices. J. Berry Res..

[22] Marquez A., Serratosa M.P., Merida J. (2014). Influence of bottle storage time on colour, phenolic composition and sensory properties of sweet red wines. Food Chem..

[23] Gómez Gallego M.A., Gómez García-Carpintero E., Sánchez-Palomo E., González Viñas M.A., Hermosín-Gutiérrez I. (2013). Evolution of the phenolic content, chromatic characteristics and sensory properties during bottle storage of red single-cultivar wines from Castilla la Mancha region. Food Res. Int..

[24] Wirth J., Caillé S., Souquet J.M., Samson A., Dieval J.B., Vidal S., Fulcrand H., Cheynier V. (2012). Impact of post-bottling oxygen exposure on the sensory characteristics and phenolic composition of Grenache rosé wines. Food Chem..

[25] Liguori G., D’Aquino S., Sortino G., de Pasquale C., Inglese P. (2015). Effects of passive and active modified atmosphere packaging conditions on quality parameters of minimally processed table grapes during cold storage. J. Berry Res..

[26] Howard L.R., Brownmiller C., Prior R.L. (2014). Improved color and anthocyanin retention in strawberry puree by oxygen exclusion. J. Berry Res..

[27] Monagas M., Gómez-Cordovés C., Bartolomé B. (2005). Evolution of polyphenols in red wines from *Vitis vinifera* L. During aging in the bottle. Eur. Food Res. Technol..

[28] Lopes P., Richard T., Saucier C., Teissedre P.-L., Monti J.-P., Glories Y. (2007). Anthocyanone A:  A quinone methide derivative resulting from malvidin 3-*O*-glucoside degradation. J. Agric. Food Chem..

[29] Mistry T.V., Cai Y., Lilley T.H., Haslam E. (1991). Polyphenol interactions. Part 5. Anthocyanin co-pigmentation. J. Chem. Soc. Perkin Trans. 2.

[30] De Beer D., Joubert E., Gelderblom W.C.A., Manley M. (2002). Phenolic compounds: A review of their possible role as *in vivo* antioxidants. S. Afr. J. Enol Vitic..

[31] Kallithraka S., Salacha M.I., Tzourou I. (2009). Changes in phenolic composition and antioxidant activity of white wine during bottle storage: Accelerated browning test *versus* bottle storage. Food Chem..

[32] Castellari M., Matricardi L., Arfelli G., Galassi S., Amati A. (2000). Level of single bioactive phenolics in red wine as a function of the oxygen supplied during storage. Food Chem..

[33] Mateus N., Carvalho E., Carvalho A.R.F., Melo A., González-Paramás A.M., Santos-Buelga C., Silva A.M.S., de Freitas V. (2003). Isolation and structural characterization of new acylated anthocyanin-vinyl-flavanol pigments occurring in aging red wines. J. Agric. Food Chem..

[34] Mateus N., Silva A.M.S., Santos-Buelga C., Rivas-Gonzalo J.C., de Freitas V. (2002). Identification of anthocyanin-flavanol pigments in red wines by NMR and mass spectrometry. J. Agric. Food Chem..

[35] He F., Liang N.N., Mu L., Pan Q.H., Wang J., Reeves M.J., Duan C.Q. (2012). Anthocyanins and their variation in red wines II. Anthocyanin derived pigments and their color evolution. Molecules.

[36] Gao Y., Tian Y., Liu D., Li Z., Zhang X.X., Li J.M., Huang J.H., Wang J., Pan Q.H. (2015). Evolution of phenolic compounds and sensory in bottled red wines and their co-development. Food Chem..

[37] Han F.L., Zhang W.N., Pan Q.H., Zheng C.R., Chen H.Y., Duan C.Q. (2008). Principal component regression analysis of the relation between CIELAB color and monomeric anthocyanins in young Cabernet Sauvignon wines. Molecules.

[38] He F., Liang N.N., Mu L., Pan Q.H., Wang J., Reeves M.J., Duan C.Q. (2012). Anthocyanins and their variation in red wines I. Monomeric anthocyanins and their color expression. Molecules.

[39] Monagas M., Núñez V., Bartolomé B., Gómez-Cordovés C. (2003). Anthocyanin-derived pigments in Graciano, Tempranillo, and Cabernet Sauvignon wines produced in Spain. Am. J. Enol. Vitic..

[40] Monagas M., Bartolomé B. (2009). Anthocyanins and anthocyanin-derived compounds. Wine Chemistry and Biochemistry.

[41] Figueiredo-Gonzalez M., Regueiro J., Cancho-Grande B., Simal-Gandara J. (2014). Garnacha tintorera-based sweet wines: Detailed phenolic composition by HPLC/DAD-ESI/MS analysis. Food Chem..

[42] Arnous A., Makris D.P., Kefalas P. (2001). Effect of principal polyphenolic components in relation to antioxidant characteristics of aged red wines. J. Agric. Food Chem..

[43] Niu Y., Zhang X., Xiao Z., Song S., Jia C., Yu H., Fang L., Xu C. (2012). Characterization of taste-active compounds of various cherry wines and their correlation with sensory attributes. J. Chromatogr. B..

[44] Pantelić M., Dabić D., Matijašević S., Davidović S., Dojčinović B., Milojković-Opsenica D., Natić M. (2014). Chemical characterization of fruit wine made from Oblačinska sour cherry. Sci. World J..

[45] Sun S.Y., Jiang W.G., Zhao Y.P. (2011). Evaluation of different *Saccharomyces cerevisiae* strains on the profile of volatile compounds and polyphenols in cherry wines. Food Chem..

[46] Xiao Z., Fang L., Niu Y., Yu H. (2015). Effect of cultivar and variety on phenolic compounds and antioxidant activity of cherry wine. Food Chem..

[47] Davidović S.M., Veljović M.S., Pantelić M.M., Baošić R.M., Natić M.M., Dabić D.Č., Pecić S.P., Vukosavljević P.V. (2013). Physicochemical, antioxidant and sensory properties of peach wine made from redhaven cultivar. J. Agric. Food Chem..

[48] Ye M., Yue T., Yuan Y. (2014). Evolution of polyphenols and organic acids during the fermentation of apple cider. J. Sci. Food Agric..

[49] Figueiredo-Gonzalez M., Cancho-Grande B., Simal-Gandara J., Teixeira N., Mateus N., de Freitas V. (2014). The phenolic chemistry and spectrochemistry of red sweet wine-making and oak-aging. Food Chem..

[50] Darias-Martin J., Martin-Luis B., Carrillo-Lopez M., Lamuela-Raventos R., Diaz-Romero C., Boulton R. (2002). Effect of caffeic acid on the color of red wine. J. Agric. Food Chem..

[51] Schwarz M., Wabnitz T.C., Winterhalter P. (2003). Pathway leading to the formation of anthocyanin-vinylphenol adducts and related pigments in red wines. J. Agric. Food Chem..

[52] Cejudo-Bastante M.J., Hermosin-Gutierrez I., Castro-Vazquez L.I., Perez-Coello M.S. (2011). Hyperoxygenation and bottle storage of Chardonnay white wines: Effects on color-related phenolics, volatile composition, and sensory characteristics. J. Agric. Food Chem..

[53] Zhang B., Liu R., He F., Zhou P.-P., Duan C.-Q. (2015). Copigmentation of malvidin-3-o-glucoside with five hydroxybenzoic acids in red wine model solutions: Experimental and theoretical investigations. Food Chem..

[54] Castillo-Muñoz N., Gómez-Alonso S., García-Romero E., Hermosín-Gutiérrez I. (2007). Flavonol profiles of *Vitis vinifera* red grapes and their single-cultivar wines. J. Agric. Food Chem..

[55] Lätti A.K., Jaakola L., Riihinen K.R., Kainulainen P.S. (2010). Anthocyanin and flavonol variation in bog bilberries (*Vaccinium uliginosum* L.) in Finland. J. Agric. Food Chem..

[56] Primetta A., Karppinen K., Riihinen K., Jaakola L. (2015). Metabolic and molecular analyses of white mutant *Vaccinium* berries show down-regulation of MYBPA1-type R2R3 MYB regulatory factor. Planta.

[57] Cejudo-Bastante M.J., Pérez-Coello M.S., Hermosín-Gutiérrez I. (2011). Effect of wine micro-oxygenation treatment and storage period on colour-related phenolics, volatile composition and sensory characteristics. LWT-Food Sci. Technol..

[58] Lago-Vanzela E.S., Procópio D.P., Fontes E.A.F., Ramos A.M., Stringheta P.C., Da-Silva R., Castillo-Muñoz N., Hermosín-Gutiérrez I. (2014). Aging of red wines made from hybrid grape cv. Brs violeta: Effects of accelerated aging conditions on phenolic composition, color and antioxidant activity. Food Res. Int..

[59] De Freitas V., Mateus N. (2006). Chemical transformations of anthocyanins yielding a variety of colours (review). Environ. Chem Lett..

[60] Ortega-Heras M., González-Sanjosé M.L. (2009). Binding capacity of brown pigments present in special Spanish sweet wines. LWT-Food Sci Technol..

[61] Martínez J.A., Melgosa M., Pérez M.M., Hita E., Negueruela A.I. (2001). Note. Visual and instrumental color evaluation in red wines. Food Sci. Technol. Int..

[62] (2006). Analytical Methods of Wine and Fruit Wine.

[63] Lee J., Durst R.W., Wrolstad R.E. (2005). Determination of total monomeric anthocyanin pigment content of fruit juices, beverages, natural colorants, and wines by the pH differential method: Collaborative study. J. AOAC Int..

[64] OIV O. (2009). Compendium of International Methods of Wine and Must Analysis.

[65] Ayala F., Echávarri J.F., Negueruela A.I. (1997). A new simplified method for measuring the color of wines. I. Red and rosé wines. Am. J. Enol. Vitic..

[66] Ayala F., Echávarri J.F., Negueruela A.I. (1999). A new simplified method for measuring the color of wines. III. All wines and brandies. Am. J. Enol. Vitic..

